# COVID-19 patients managed in psychiatric inpatient settings due to first-episode mental disorders in Wuhan, China: clinical characteristics, treatments, outcomes, and our experiences

**DOI:** 10.1038/s41398-020-01022-x

**Published:** 2020-10-02

**Authors:** Qin Xie, Fang Fan, Xue-Peng Fan, Xiao-Jiang Wang, Ming-Jian Chen, Bao-Liang Zhong, Helen Fung-Kum Chiu

**Affiliations:** 1 Department of Psychiatry, Wuhan Mental Health Center, Wuhan, Hubei China; 2 Affiliated Wuhan Mental Health Center, Tongji Medical College of Huazhong University of Science & Technology, Wuhan, Hubei China; 3 Department of Critical Care Medicine, The First Hospital of Wuhan Municipality, Wuhan, Hubei China; 4 Department of Respiratory Medicine, Affiliated Hospital of Jianghan University, Wuhan, Hubei China; 5 Research Center for Psychological and Health Sciences, China University of Geosciences, Wuhan, Hubei China; 6 Department of Psychiatry, The Chinese University of Hong Kong, Hong Kong SAR, China

**Keywords:** Schizophrenia, Depression

## Abstract

Data are scarce regarding the comorbid mental disorders and their management among COVID-19 patients. This study described the clinical characteristics and management of COVID-19 patients treated in psychiatric inpatient settings due to comorbid first-onset mental disorders in Wuhan, China. This electronic medical records-based study included 25 COVID-19 patients with first-onset mental disorders and 55 patients with first-onset mental disorders without COVID-19 (control group). Data collected included ICD-10 diagnoses of mental disorders, psychiatric and respiratory symptoms, treatments, and outcomes. Adjustment disorder (*n* = 11, 44.0%) and acute and transient psychotic disorders, with associated acute stress (*n* = 6, 24.0%) were main clinical diagnoses in the COVID-19 group while serious mental illnesses (i.e., schizophrenia, 24.5%) and alcohol use disorders (10.9%) were overrepresented in the control group. On admission, the most common psychiatric symptom in COVID-19 patients was insomnia symptoms (*n* = 18, 72.0%), followed by aggressive behaviors (*n* = 16, 64.0%), delusion (*n* = 10, 40.0%), and severe anxiety (*n* = 9, 36.0%). In addition to respiratory treatments, 76.0% COVID-19 patients received antipsychotics, 40.0% sedative-hypnotics, and 24.0% mood stabilizers. At the end of inpatient treatment, 4 (16.0%) COVID-19 patients were transferred to other hospitals to continue respiratory treatment after their psychiatric symptoms were controlled while the remaining 21 (84.0%) all recovered. Compared to the control group, COVID-19 group had significantly shorter length of hospital stay (21.2 vs. 37.4 days, *P* < 0.001). Adjustment disorder and acute and transient psychotic disorders are the main clinical diagnoses of COVID-19 patients managed in psychiatric inpatient settings. The short-term prognosis of these patients is good after conventional psychotropic treatment.

## Introduction

Globally, the ongoing COVID-19 pandemic has posed unprecedented challenges to both the mental health of affected people and mental healthcare system of affected countries^1^. In nonclinical settings, studies have reported the high prevalence of depressive symptoms, anxiety symptoms, and post-traumatic stress disorder (PTSD) symptoms and their characteristics in a variety of COVID-19 epidemic affected populations, including general populations, university students, and health workers^2–6^. Two clinical studies, one conducted in the isolation treatment ward of a general hospital (*n* = 106) and the other in temporary quarantine facilities (*n* = 714) in Wuhan, China, reported that as high as 9.4%, 15.1%, 24.5%, and 96.2% of the COVID-19 patients had severe depressive symptoms, severe anxiety symptoms, suicidal ideation, and clinically significant PTSD symptoms, respectively^7,8^. These findings suggest that COVID-19 patients are at greater risk for developing mental disorders. However, studies focusing on COVID-19 patients who are sufficiently impaired to justify a clinical diagnosis of mental disorder still remain very limited^9^.

Findings from the clinical studies in samples of SARS-infected and MERS-infected patients have shown that various psychiatric and neuropsychiatric presentations are commonly seen during both the acute phase of infection and the postinfection stage^9^. For example, in China, among SARS-infected patients 10.4% had disturbance of consciousness, 4.6% psychotic disorders, and 30.6% mood disorders while among SARS survivors assessed at a median follow-up time of 41.3 months since the infection 54.5% had PTSD, 39.0% depressive disorders, 36.4% somatoform pain disorder, 32.5% panic disorder, and 15.6% obsessive compulsive disorder^10,11^. In a case series of ten SARS-infected patients who were referred for Consultation and Liaison Psychiatry services, five patients were diagnosed with adjustment disorder, two organic hallucinosis, and two organic manic disorder^12^. Accordingly, the “Principles for Emergency Psychological Crisis Intervention for COVID-19 Pneumonia Epidemic”, released by the National Health Commission of China on 27th January, 2020, has defined COVID-19 patients as the group most in need of psychiatric treatment and psychological intervention^13^. Nevertheless, managing COVID-19 patients with mental disorders is undoubtedly challenging for psychiatrists because of their insufficient training in treating infectious diseases and the complex clinical presentations of COVID-19 patients having comorbidity of mental disorders.

A greater understanding on the psychopathology of COVID-19 patients could facilitate their clinical management. Early identification and effective treatment of co-existing mental disorders could also improve outcomes of respiratory treatment. As far as we know, until now, only two studies have examined mental disorders in COVID-19 patients: in United States, 4.6, 3.8, and 3.4% of the patients had anxiety and other related disorders, mood disorders, and sleep disorders, respectively^14^, and, in United Kingdom, 8.0, 4.8, and 3.2% of the patients had psychosis, dementia-like syndrome, and mood disorders, respectively^15^. However, both studies did not provide data regarding clinical characteristics, management, and outcomes of COVID-19 patients with comorbid mental disorders. Given this knowledge gap in psychiatric management of COVID-19, the present study described characteristics, treatments, and outcomes of 25 COVID-19 patients with first-episode mental disorders treated in a designated psychiatric hospital in Wuhan, China. As front-line mental health workers, we also shared some experiences in managing these patients. In this study, inpatients without COVID-19 presenting with first-episode mental disorders during the period of COVID-19 outbreak were recruited as a control group. As most COVID-19 patients have no past history of mental disorders, the control group included only patients with first-onset mental disorders.

## Methods

### Study design and subjects

This was a single-center, real-world, retrospective, and observational study. Subjects were new patients with first-episode mental disorders who were admitted to the inpatient department of Wuhan Mental Health Center from 23rd January to 19th April, 2020, a period spanning the COVID-19 epidemic outbreak in Wuhan, China. Wuhan Mental Health Center is the largest public psychiatric hospital in central south region of China with approximately 1200 inpatient beds, and provides outpatient, inpatient, and community mental health services to the over 10 million residents of Wuhan and its surrounding regions. Since the COVID-19 outbreak in Wuhan, the hospital has served as the only designated hospital for treating both psychiatric patients infected with SARS-CoV-2 and COVID-19 patients with mental disorders in Hubei province. Because COVID-19 patients with new onset of mental disorders are different from psychiatric patients infected with SARS-CoV-2 and most COVID-19 patients in real-world clinical practice have no mental disorders before the infection, we did not include SARS-CoV-2-infected psychiatric patients as subjects. The inclusion criteria for this study were: 1) no previous psychiatric disorders, 2) presentation at our hospital for the first time, 3) a diagnosis of mental disorder according to ICD-10 criteria for mental and behavioral disorders, 4) admission in our hospital during the COVID-19 outbreak period, and 5) having accurate SARS-CoV-2 RNA test result to indicate the presence or absence of COVID-19, either before or after hospitalization. Suspected COVID-19 patients and psychiatric patients with a hospital-acquired SARS-CoV-2 infection were excluded. After a detailed review of medical records of a total of 753 psychiatric patients during that period, 80 eligible patients were identified: 25 with and 55 without laboratory-confirmed SAR-CoV-2 infection.

This study was approved by the Ethics Committee of Wuhan Mental Health Center (approval number: KY2020.01.06). Informed consent from patients was waived by the committee because personal identifying information had been removed from the electronic medical records before our analysis.

### Procedures and assessments

Two experienced clinicians, one psychiatrist and one pulmonologist, worked together to extract data by using a standardized case-report form. Before the formal study, they were trained on how to collect data from electronic medical records and patients and their treating psychiatrists (when necessary). The following data were collected from patients’ admission dates until 10th May, 2020.

1. Clinical data
  - Socio-demographic characteristics: age, gender, marital status, education, occupation (unemployed, front-line workers, and others), self-rated economic status (poor, moderate, good), and identity (Wuhan residents vs. visitors of Wuhan). Front-line workers were individuals who had direct contact with SARS-CoV-2-positive persons or individuals with suspected SARS-CoV-2 infection, including physicians, nurses, staff of centers for disease prevention and control, and policemen.
  - Psychiatric data: These included ICD-10 diagnosis of mental disorder at discharge, family history of mental disorder, precipitating circumstances of mental disorder, mode of hospital admission (voluntary, involuntary, and transferred from temporary quarantine facilities and other hospitals), physical comorbidity, and psychiatric symptoms on admission.
  - Respiratory data: These included the severity of COVD-19 on admission^16^, as well as the respiratory symptoms on admission: fever (body temperature ≥37.5 °C), cough, fatigue, shortness of breath, myalgia, chills, and headache.
  - Radiological findings on admission: the presence and distribution (unilateral vs. bilateral) of abnormalities on chest CT.
  - Laboratory findings on admission: counts of white blood cells, neutrophils, and lymphocytes, and C-reactive protein (CRP).
  - Psychiatric medications used: antidepressants, anti-anxiety medications, mood-stabilizing medications, sedative-hypnotics, and antipsychotic medications.
  - Respiratory treatment: antibiotics, antiviral drugs, oxygen therapy, and mechanical ventilation.
2. Outcomes
  - Psychiatric outcomes: As a routine clinical practice for research purpose in our hospital, inpatients were assessed weekly with psychiatric symptom scales to monitor treatment response. Accordingly, we extracted Hamilton Rating Scale for Depression, 17 item version (HRSD-17), Hamilton Rating Scale for Anxiety (HRSA) scores, and Positive and Negative Syndrome Scale (PANSS) scores for COVID-19 patients. In general, a HRSD-17 score of 7 or lower, a HRSA score of 7 or lower, and a PANSS total score of 58 or lower were used denote no depressive symptoms, no anxiety symptoms, and mildly ill in clinical severity of psychotic disorders^17,18^.
  - Respiratory outcomes: complications during treatment, results of SARS-CoV-2 RNA and antibody tests at discharge, and body temperature.
  - Composite outcomes. The primary composite outcome of inpatient treatment included discharge, transfer to other hospitals, and death. Because of the suspension of public transportations within Hubei province, some patients were held up in our hospital despite having met criteria for discharge, as indicated in the medical records. The outcome “discharge” also included “having met criteria for discharge”. The secondary composite outcome was length of hospital stay, defined as days from admission to discharge. Treatment-related adverse events during inpatient treatment were also collected.

### Management of COVID-19 patients

**Fig. 1:**
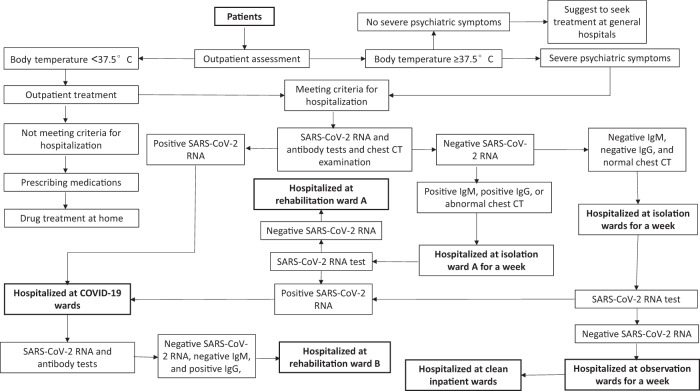
Standardized operation procedures for screening and admitting patients and measures adopted for the prevention of SARS-CoV-2 infection in inpatient wards. Note: measures adopted at inpatient wards during the COVID-19 outbreak: **a** Medical staff of COVID-19 wards, isolation wards, rehabilitation wards, observation wards, and general wards must stay at the hospital during working hours and live in the designated hotels during resting time. Returning home, face-to-face contacting with any people, and going to places other than the hospital and designated hotels were not allowed; **b** Leave from hospital (e.g., day leave and home leave) and routine family visits were not allowed for patients of all wards. No outside caregiver was permitted to care for the patients in COVID-19 wards and isolation wards. SARS-CoV-2 negative caregivers were allowed to care for patients in rehabilitation wards, observation wards, and general wards, however, they must always stay at the wards; **c** Body temperature was measured twice daily for all patients and all medical staff; **d** Medical staff were regularly trained on the prevention, diagnosis, and treatment of COVID-19; **e** Medical staff received SARS-CoV-2 RNA and antibody tests twice a month. Medical staff must wear personal protective equipment when interviewing or in direct contact with patients.

At the beginning of the COVID-19 outbreak, we established a new standardized operation procedure to screen and admit patients according to their levels of SARS-CoV-2 infection (confirmed, suspected, and clean). Special wards were set up: COVID-19 wards for COVID-19 patients, isolation wards for patients with suspected COVID-19, and observation wards for patients with negative results on SARS-CoV-2 RNA and antibody tests, and normal chest CT. Strict measures for preventing the nosocomial infection of SARS-CoV-2 among medical staff and patients were also implemented. Figure 1 depicts the flowchart of patients admission and prevention measures adopted in different wards.

Moderate, severe, and critical COVID-19 patients were treated by psychiatrists of the Psychiatric Intensive Care Unit (PICU) of our hospital, under the co-supervision of two experienced specialists in respiratory medicine and critical care medicine, respectively. Unlike PICUs in psychiatric hospitals of most countries, our PICU was set up in 2018 and provides inpatient services for psychiatric patients with major medical conditions. It has all necessary medical equipments for providing basic lifesaving services such as electrocardiogram monitors, ventilators, defibrillators, and blood purification equipments.

### Statistical analysis

We described the characteristics of COVID-19 patients and compared them with those of the control group. For testing group differences, Chi-square test or Fisher’s exact test was used for categorical variables, independent-sample *t*-test for continuous variables of normal distribution, and Mann–Whitney *U*-test for continuous variables of skewed distribution. The statistical significance level was set at *P* < 0.05 (two-sided). All data analyses were conducted with SPSS 15.0.

## Results

### Sociodemographic characteristics of COVID-19 patients with mental disorders

The COVID-19 group had 13 men (52.0%), with an average age of 53.1 years (standard deviation [SD]: 12.9). Compared to the control group, COVID-19 patients were significantly more likely to be older (median: 50 vs. 41 years), be married (76.0 vs. 32.7%), have an education level of college and above (50.0 vs. 23.6%), be front-line workers (44.0 vs. 7.3%), and have a good economic status (44.0 vs. 1.8%) (*P* ≤ 0.041) (Table 1).

**Table 1:** Sociodemographic characteristics of patients without and with COVID-19 presenting with first-episode mental disorders, *n* (%).

Variables		NonCOVID-19 patients (*n* = 55)	COVID-19 patients (*n* = 25)	*χ*^2^/*t*	*P*
Gender	Male	33 (60.0)	13 (52.0)		
	Female	22 (40.0)	12 (48.0)	0.450	0.502
Age (years)	Mean ± standard deviation	40.7 ± 13.7	53.1 ± 12.9	3.764	<0.001
	Range, median	15–70, 41	33–86, 50		
Marital status	Married	18 (32.7)	19 (76.0)		
	Divorced/widowed	12 (21.8)	3 (12.0)		
	Single	25 (45.5)	3 (12.0)	13.338	0.001
Education level	Primary school and below	12 (21.8)	4 (16.0)		
	Middle school	30 (54.5)	8 (32.0)		
	College and above	13 (23.6)	13 (50.0)	6.385	0.041
Occupation	Unemployed	40 (72.7)	9 (36.0)		
	Front-line workers	4 (7.3)	11 (44.0)		
	Others	11 (20.0)	5 (20.0)	16.150	<0.001
Identity	Wuhan residents	46 (83.6)	23 (92.0)		
	Visitors of Wuhan	9 (16.4)	2 (8.0)		0.488^a^
Self-rated economic status	Poor	19 (34.5)	3 (12.0)		
	Moderate	35 (63.6)	11 (44.0)		
	Good	1 (1.8)	11 (44.0)	24.717	<0.001

^a^Fisher’s exact test.

### Psychiatric clinical characteristics and treatment of COVID-19 patients with mental disorders

The clinical diagnoses of the COVID-19 group at discharge were mainly consisted of adjustment disorder (14.0%) as well as acute and transient psychotic disorders, with associated acute stress (24.0%) while those of the control group were mainly consisted of schizophrenia (24.5%), acute and transient psychotic disorders, without associated acute stress (23.6%), mood disorders (20.0%), and alcohol use disorders (10.9%). No patients in the COVID-19 group were diagnosed with mood disorders and alcohol use disorders (Table 2). On admission, the most common psychiatric symptom of the COVID-9 group was insomnia symptoms (72.0%), followed by aggressive behaviors (64.0%), delusion (40.0%), and severe anxiety symptoms (36.0%) while the top four common symptoms of the control group were insomnia symptoms (63.6%), aggressive behaviors (60.0%), delusion (50.9%), and abnormally elevated mood (47.3%). In addition, in comparison to the control group, COVID-19 group was significantly more likely to ascribe the onset of mental disorders to COVID-19 per se and its treatments (92.0% vs. 32.7%) and be referred from other institutions (44.0% vs. 1.8%) (*P* < 0.001) (Table 3).

**Table 2:** ICD-10 clinical diagnoses of mental disorders at discharge of patients with and without COVID-19 presenting with first-onset mental disorders, *n* (%).

Diagnosis	NonCOVID-19 patients (*n* = 55)	COVID-19 patients (*n* = 25)	*χ*^2^	*P*
Mood disorders	12 (20.0)	0 (0.0)		0.014*
Manic episode, severe with psychotic symptoms	4 (7.3)	0 (0.0)		
Manic episode without psychotic symptoms	2 (3.6)	0 (0.0)		
Bipolar disorder, current episode depressed, severe, without psychotic features	1 (1.8)	0 (0.0)		
Bipolar disorder, current episode depressed, severe, with psychotic features	1 (1.8)	0 (0.0)		
Bipolar disorder, current episode manic severe with psychotic features	1 (1.8)	0 (0.0)		
Major depressive disorder, single episode, severe with psychotic features	3 (5.5)	0 (0.0)		
Alcohol use disorders	6 (10.9)	0 (0.0)		0.096^a^
Mental and behavioral disorders due to use of alcohol, acute intoxication	4 (7.3)	0 (0.0)		
Mental and behavioral disorders due to use of alcohol, psychotic disorder	1 (1.8)	0 (0.0)		
Alcohol dependence	1 (1.8)	0 (0.0)		
Psychotic disorders	35 (63.6)	11 (44.0)	2.712	0.101
Delirium not superimposed on dementia	0 (0.0)	2 (8.0)		
Acute and transient psychotic disorders, with associated acute stress	7 (12.7)	6 (24.0)		
Acute and transient psychotic disorders, without associated acute stress	13 (23.6)	0 (0.0)		
Schizophrenia	14 (24.5)	1 (4.0)		
Organic hallucinosis	0	1 (4.0)		
Chloroquine-induced psychosis	0	1 (4.0)		
Trance and possession disorders	1 (1.8)	0 (0.0)		
Anxiety disorders	2 (3.6)	14 (56.0)	29.455	<0.001
Acute stress reaction	0 (0.0)	2 (8.0)		
Panic disorder	0 (0.0)	1 (4.0)		
Adjustment disorder, mixed anxiety and depressive reaction	1 (1.8)	11 (44.0)		
Organic anxiety disorder	1 (1.8)	0 (0.0)		

^a^Fisher’s exact test.

**Table 3:** Clinical characteristics, results of radiological and laboratory examinations, and psychiatric medications used of patients with and without COVID-19 presenting with first-episode mental disorders, *n* (%).

Variables		NonCOVID-19 patients (*n* = 55)	COVID-19 patients (*n* = 25)	*χ*^2^	*P*
Family history of mental disorders	Positive	8 (14.5)	3 (12.0)		0.531^a^
Precipitating circumstances of mental disorders	None	33 (60.0)	2 (8.0)		
	COVID-19 related	18 (32.7)	23 (92.0)		
	Others	4 (7.3)	0 (0.0)	24.970	<0.001^a^
Mode of hospital admission	Voluntary	19 (34.5)	14 (58.3)		
	Involuntary	35 (63.6)	0 (0.0)		
	Transferred from other institutions	1 (1.8)	11 (44.0)	42.913	<0.001^a^
Major medical condition	Presence	19 (34.5)	11 (44.0)	0.656	0.418
Diabetes mellitus	Presence	3 (5.5)	4 (16.0)		0.196^a^
Hypertension	Presence	8 (14.5)	9 (36.0)	4.728	0.030
Psychiatric symptoms on admission	Hallucination	22 (40.0)	8 (32.0)	0.469	0.493
	Delusion	28 (50.9)	10 (40.0)	0.820	0.365
	Abnormally elevated mood	26 (47.3)	5 (20.0)	5.386	0.020
	Severe depressive symptoms	6 (10.9)	6 (24.0)	2.310	0.129
	Fear	13 (23.6)	5 (20.0)	0.130	0.718
	Severe anxiety symptoms	18 (32.7)	9 (36.0)	0.082	0.774
	Self-harm or attempted suicide	7 (12.7)	1 (4.0)		0.424^a^
	Aggressive behaviors	33 (60.0)	16 (64.0)	0.116	0.734
	Running out of home	8 (14.5)	0 (0.0)		0.052^a^
	Disorientation	5 (9.1)	0 (0.0)		0.318^a^
	Insomnia symptoms	35 (63.6)	18 (72.0)	0.538	0.463
Chest CT on admission	Normal	52 (94.5)	6 (24.0)		
	Abnormal	3 (5.5)	19 (76.0)	42.902	<0.001
	Unilateral	1 (1.8)	4 (16.0)		
	Bilateral	2 (3.6)	15 (60.0)		
Count of white blood cells (10^9^/L)	<4	3 (5.5)	4 (16.0)		
	4–10	42 (76.4)	18 (72.0)		
	>10	10 (18.2)	3 (12.0)	2.517	0.294^a^
Count of neutrophils (10^9^/L)	<2	1 (1.8)	3 (12.0)		
	2–7	43 (78.2)	19 (76.0)		
	>7	11 (20.0)	3 (12.0)	3.760	0.146^a^
Count of lymphocytes (10^9^/L)	<0.8	3 (5.5)	3 (12.0)		
	0.8–4.0	52 (94.5)	22 (88.0)		0.370^a^
C-reactive protein (mg/L)	<8	45 (81.8)	10 (40.0)		
	≥8	10 (18.2)	15 (60.0)	13.990	<0.001
Psychiatric medications used	Antipsychotics	49 (89.1)	19 (76.0)	2.310	0.129
	Olanzapine	30 (54.5)	12 (48.0)	0.295	0.587
	Mood stabilizers	23 (41.8)	6 (24.0)	2.361	0.124
	Sedative-hypnotics	21 (38.2)	10 (40.0)	0.024	0.877
	Antidepressants	7 (12.7)	4 (16.0)	0.155	0.694
	Anti-anxiety medications	1 (1.8)	1 (4.0)		0.530^a^

^a^Fisher’s exact test.

The two most commonly proscribed psychotropic medications were antipsychotics (76.0%) and sedative-hypnotics (40.0%) in the COVID-19 group and antipsychotics (89.1%) and mood stabilizers (41.8%) in the control group. Olanzapine was the most often prescribed antipsychotics: 48.0% in the COVID-19 group and 54.5% in the control group (Table 3).

### Respiratory clinical characteristics, complications, and treatment of COVID-19 patients with mental disorders

Among the COVID-19 patients, 14 (56.0%) and 5 (20.0%) had moderate and severe-to-critical COVID-19, respectively. The most common respiratory symptom on admission was cough (88.0%), followed by fever (56.0%) and chills (48.0%). All patients received intravenous antibiotic therapy and antiviral therapy (100% arbidol); oxygen therapy and noninvasive mechanical ventilation were administered to 28.0% and 8.0% of the patients, respectively. During the hospital admission, 76.0% patients received a diagnosis of pneumonia, followed by acute respiratory distress syndrome (12.0%) and acute kidney injury (8.0%) (Table 4). 19 (76.0%) patients had abnormalities on chest CT: 16.0% unilateral and 60.0% bilateral. On admission, leukopenia was present in 16.0% of the patients, lymphocytopenia in 12.0%, and elevated levels of CRP in 60.0% (Table 3).

**Table 4:** Respiratory characteristics, treatment, and outcomes of COVID-19 patients presenting with first-episode mental disorders.

Variables		*n*	%
COVID-19 severity	Mild	4	16.0
	Moderate	14	56.0
	Severe	5	20.0
	Critical	2	8.0
Symptoms on admission	Fever	14	56.0
	Cough	22	88.0
	Shortness of breath	7	28.0
	Fatigue	10	40.0
	Myalgia	4	16.0
	Chills	12	48.0
	Headache	4	16.0
Treatment	Intravenous antibiotics	25	100
	Oseltamivir	7	28.0
	Chloroquine	5	20.0
	Arbidol	25	100.0
	Methylprednisolone	5	20.0
	Oxygen therapy	7	28.0
	Noninvasive mechanical ventilation	2	8.0
Complications during hospitalization	Septic shock	1	4.0
	Acute respiratory distress syndrome	3	12.0
	Acute kidney injury	2	8.0
	Rhabdomyolysis	1	4.0
	Pneumonia	19	76.0
SARS-CoV-2 RNA test at discharge	Negative	22	88.0
SARS-CoV-2 antibody test at discharge	IgM negative	16	64.0
	IgG positive	17	68.0

### Outcomes of inpatient treatment of COVID-19 patients with mental disorders

**Fig. 2:**
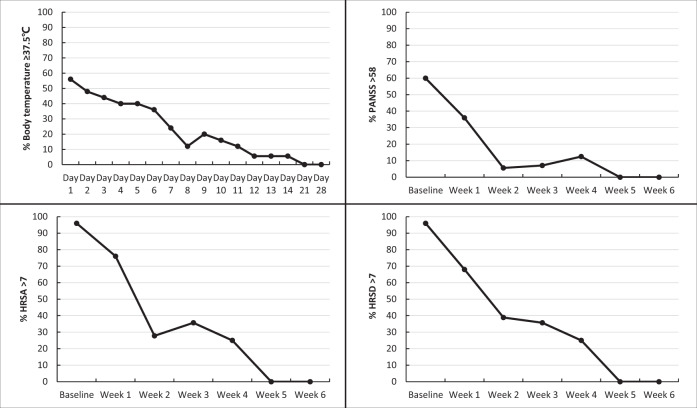
Time trends of percentages of body temperature ≥ 37.5 °C, PANSS score > 58, HRSA Score > 7, and HRSD score > 7 among COVID-19 patients during the hospitalization. PANSS positive and negative syndrome scale, HRSA Hamilton rating scale for anxiety, *HRSD* Hamilton rating scale for depression.

At the end of inpatient treatment, 4 (16.0%) COVID-19 patients were transferred to other hospitals to continue their respiratory treatment after psychiatric symptoms were successfully resolved and the remaining 21 patients (84.0%) were discharged or satisfied the criteria for discharge. 22 (88.0%) patients were negative on SARS–CoV-2 RNA test, 64.0% negative on IgM test, and 68.0% positive on IgG test. Figure 2 shows that only 12.0% of the COVID-19 patients had fever on the 8th day of the treatment, and only 5.6% had PANSS score above the threshold value for “mild psychotic disorders”. Proportions of patients having depressive and anxiety symptoms both decreased from over 90.0% at baseline to 25.0% at the 4th week, and further decreased to 0 thereafter. The COVID-19 group had significantly shorter length of hospital stay than the control group (21.2 vs. 37.4 days, *P* < 0.001) (Tables 4 and 5).

**Table 5:** Treatment outcomes of patients with and without COVID-19 presenting with mental disorders, *n* (%).

Variables		NonCOVID-19 patients (*n* = 55)	COVID-19 patients (*n* = 25)	*χ*^2^/*t*	*P*
Primary composite outcome	Discharge	55 (100.0)	21 (84.0)		
	Transfer to other hospitals	0 (0.0)	4 (16.0)		0.008^a^
	Death	0 (0.0)	0 (0.0)		
Secondary composite outcome	Length of hospital stay (days)	37.4 ± 15.5	21.2 ± 13.4	4.517	<0.001

^a^Fisher’s exact test.

During the treatment, the most common adverse events of the COVID-19 group were nausea (12.0%), diarrhea (12.0%), liver dysfunction (12.0%), and tachycardia (12.0%) while those of the control group were weight gain (1.7%), tachycardia (10.9%), drowsiness (7.3%), and akathisia (7.3%) (Table 6).

**Table 6:** Adverse events of patients with and without COVID-19 presenting with mental disorders during the treatment, *n* (%).

Adverse events	NonCOVID-19 patients (*n* = 55)	COVID-19 patients (*n* = 25)	*P*
Drowsiness	4 (7.3)	1 (4.0)	1.000^a^
Nausea or vomiting	0 (0.0)	3 (12.0)	0.028^a^
Diarrhea	0 (0.0)	3 (12.0)	0.028^a^
Dizziness or syncope	1 (1.8)	2 (8.0)	0.229^a^
Anorexia or decreased appetite	0 (0.0)	2 (8.0)	0.229^a^
Weight gain	7 (12.7)	0 (0.0)	0.092^a^
Tremor	3 (5.5)	1 (4.0)	1.000^a^
Akathisia	4 (7.3)	0 (0.0)	0.304^a^
Constipation	3 (5.5)	1 (4.0)	1.000^a^
Abnormal blood routine	1 (1.8)	2 (8.0)	0.229^a^
Liver dysfunction	2 (3.6)	3 (12.0)	0.173^a^
Tachycardia	6 (10.9)	3 (12.0)	1.000^a^

^a^Fisher’s exact test.

## Discussion

During the outbreak of COVID-19, the selection of an appropriate treatment setting for COVID-19 patients with mental disorders is a dilemma: in respiratory treatment settings these patients are more likely to not adhere with the treatment, and even pose a safety threat to clinical staff and other patients, while in psychiatric treatment settings psychiatrists have limited ability to manage COVID-19 and its complications, probably worsening physical health outcomes of the patients. Our study provided the first-hand data on the clinical characteristics, treatments, and outcomes of COVID-19 patients with mental disorders treated in a psychiatric setting in China’s COVID-19 epicenter. In fact, of the 25 COVID-19 patients, no one died, and 21 had good recovery under the joint management of our collaborative treatment team. Psychiatric symptoms of the remaining four patients, although they were referred to other hospitals, were also successfully controlled. Importantly, none of the medical staff working in the COVID-19 wards were infected. Our experiences may be helpful for the clinical management of COVID-19 patients with mental disorders in other countries.

The main findings of this comparative study are 1) adjustment disorder and acute and transient psychotic disorders, with associated acute stress were the main clinical diagnoses in the COVID-19 group and some other disorders had their organic basis such as delirium due to infection and chloroquine-induced psychosis, while serious mental illnesses (SMIs) and alcohol use disorders were overrepresented in the control group, a common feature of inpatients of most Chinese psychiatric hospitals; 2) a wide range of psychiatric symptoms were found in COVID-19 patients with mental disorders on admission, including psychotic symptoms, aggressive behaviors, and anxiety symptoms; 3) the most common respiratory symptom of COVID-19 patients was cough, followed by fever, chills, and fatigue; and 4) mental disorders and COVID-19 of most patients were successfully treated after symptomatic and supportive treatments, including conventional psychotropic treatment and antiviral treatment, and, COVID-19 patients left the hospital earlier than psychiatric patients without COVID-19, on average by 16 days after admission.

Compared to the clinical characteristics of 1099 Chinese COVID-19 patients admitted to general hospitals^19^, our sample of COVID-19 patients with mental disorders had more severe COVID-19 and more respiratory symptoms but had lower rates of leukopenia, lymphocytopenia, and abnormal chest CT. The more severe COVID-19 illness may partially explain the onset of mental disorders in our sample of COVID-19 patients. Because the sample is very small in this study, it remains unclear whether the low rates of leukopenia and lymphocytopenia are unique in COVID-19 patients with mental disorders. The significantly higher level of socioeconomic status in the COVID-19 group than the control group is consistent with the low socioeconomic status of patients with SMIs^20^. This phenomenon may also be related to good short-term outcomes of inpatient treatment in the COVID-19 group.

Depression, anxiety, and fear are commonly seen in persons under any crisis situation due to a variety of stressors^21^. Suffering from COVID-19 per se, a potentially fatal disease, is a major stressor for COVID-19 patients. Isolation, separation from family members, and severe side effects of antiviral treatment are other significant sources of stress. Perhaps, because of so many unusual stressors, various stress reactions occur among COVID-19 patients, ranging from insomnia and anxiety symptoms to psychotic symptoms and aggressive behaviors. This pattern of psychiatric symptoms is similar to that of SARS-infected patients in a previous study, ranging from mild psychiatric problems such as anger, anxiety, and depressive reactions to severe psychotic problems such as hallucinatory and manic mood^12^. The disease course of COVID-19 is generally short, approximately two weeks in hospitalized patients^19,22^, so these psychiatric symptoms usually do not last long enough to meet the course criteria for mood disorders, SMIs, PTSD, and other specific disorders. As a result of this, few COVID-19 patients were diagnosed with mood disorders, PTSD, and SMIs. Accordingly, adjustment disorder and acute and transient psychotic disorders consist of the main diagnoses of mental disorders in COVID-19 patients. This finding is partly in line with results of the aforementioned study in United Kingdom^15^: psychosis and dementia-like syndrome were the two most common mental disorders in COVID-19 patients. Nonetheless, this is inconsistent with findings from the aforementioned study in United States^14^: anxiety and mood disorders were the two most common mental disorders in COVID-19 patients. These discrepancies might be due to the different patient characteristics between psychiatric inpatient settings and general hospitals.

Due to the direct central nervous system (CNS) invasion, induction of CNS inflammatory mediators, and other pathophysiologic mechanisms of SARS-CoV-2, delirium is very likely to occur in patients in the acute stage of COVID-19^23^. For example, 65% of the COVID-19 patients receiving intensive care had confusion and 69% had agitation^9^. In our sample of COVID-19 patients, only 2 (8.0%) were diagnosed with delirium. In general, delirium is often transient and can be appropriately addressed by physicians in general hospitals. Only patients with long-lasting severe agitation, destructive behaviors, and severe hallucination are likely to be referred for psychiatric treatment. Because the two patients were transferred from other hospitals, this explains the relatively lower rate of delirium in our sample.

Because there has been no effective antiviral drugs for COVID-19^24^, the basic treatment principle of COVID-19 is proactively preventing complications and progression to severe or critical illness via symptomatic and supportive treatments^25^. Accordingly, antibiotics, oxygen, physical cooling, and mechanical ventilation were provided to mitigate patients’ respiratory symptoms. Based on our clinical experiences, the antiviral drugs we used had little effect on COVID-19, rather they had obvious side effects, particularly chloroquine. As shown in Table 6, more adverse events due to antiviral drugs occurred in the COVID-19 group, including vomiting, diarrhea, dizziness, decreased appetite, and liver dysfunction. In our study, one patient was diagnosed with chloroquine-induced psychosis because her psychotic symptoms occurred shortly after chloroquine administration and disappeared very soon after stopping chloroquine. Drug-induced psychosis has been well-recognized as a side effect of chloroquine for many years, and this drug also has a lot of other side effects^26,27^, so clinicians must keep alert to the mental health status of patients prescribed with this drug.

Possibly because these patients were psychotropic-naïve before admission, they responded very well and quickly to psychotropic medications: as displayed in Fig. 2, from baseline to week 2, the proportion of patients with mild psychotic symptoms had decreased from 60.0 to 5.6%, and proportions of anxiety symptoms and depressive symptoms also substantially decreased from 96.0 to 27.8% and from 96.0 to 38.9%, respectively. In addition, doses of psychotropic medications used in the COVID-19 group were generally lower than the control group (i.e., olanzapine: 2.5–5 mg/day for combination therapy and 10–15 mg/day for monotherapy). The use of olanzapine in treating mental disorders in COVID-19 patients deserves to be emphasized despite its side effect of metabolic syndrome and obesity. Because many patients had insomnia and decreased appetite, a low-dose olanzapine alone or in combination with other medication was administered to nearly a half of the patients. Olanzapine has relatively potent sedative and appetite-increasing effects, which may be of benefit to certain patients^28^. Indeed, we did observe that patients slept well and ate well after the administration of olanzapine. We consider that psychiatric medication treatment is an essential component of the symptomatic treatment strategy for COVID-19 patients with mental disorders, even more important than respiratory treatment. This is because it facilitates adherence with the respiratory treatment and hence may lead to the earlier recovery of patients.

Finally, it is worth-noting that, despite the significantly shorter length of hospital stay in COVID-19 group than control group in our study, the length of hospital stay of COVID-19 patients with comorbid mental disorders in our study is still longer than that of COVID-19 patients in general hospitals (21.2 vs. 12.8 days)^19^. This possibly reflects the negative impact of comorbid mental disorders on the prognosis of COVID-19 patients: prolonging hospital stay and delaying the recovery, suggesting the importance of early identification and intervention for COVID-19 patients with mental disorders.

A notable limitation of our study is the small sample size of COVID-19 patients with mental disorders. Further, as we mentioned above, clinical characteristics of COVID-19 patients with mental disorders may differ significantly between psychiatric inpatient settings and general hospitals. Both limit the external validity of our findings. Second, because this is a real-world study, we did not attempt to include an age matched control group or do multiple regression analysis to adjust for this unmatched factor. In fact, the onset of SMIs such as schizophrenia and bipolar disorders are usually in young adulthood while older adults are more susceptible to COVID-19^29–31^. Hence the younger age of patients without COVID-19 presenting with first-onset mental disorders compared to COVID-19 patients with first-onset mental disorders is expected. Third, we did not follow-up these patients after discharge, so the long-term prognosis of COVID-19 patients with mental disorders warrants further studies. Fourth, the involvement of PICU in our treatment model is unique, limiting the generalizability of our findings. Nevertheless, since it is uncertain how long COVID-19 will remain a major problem, and there may be other disease outbreak or pandemics in future, the model of PICU may be one useful option. Finally, electroencephalogram and CSF examinations were not performed, so it remains uncertain if psychiatric symptoms of COVID-19 patients are COVID-19-induced CNS manifestations.

In summary, stress-related disorders such as adjustment disorder as well as acute and transient psychotic disorders are the main clinical diagnoses of COVID-19 patients with mental disorders. These patients have a variety of psychiatric presentations ranging from depressive and anxiety symptoms to psychotic symptoms. Nevertheless, their short-term prognosis is good after conventional psychotropic treatment. Because there is no effective antiviral treatment, symptomatic and supportive treatments, in particular psychiatric medication therapy, are important for the recovery of COVID-19 patients with mental disorders. In addition, our experiences suggest that the administration of a low-dose olanzapine seems helpful for the clinical management of these patients.
